# Characterizing the inflammatory response in esophageal mucosal biopsies in children with eosinophilic esophagitis

**DOI:** 10.1038/cti.2016.30

**Published:** 2016-07-01

**Authors:** Wael N Sayej, Antoine Ménoret, Anu S Maharjan, Marina Fernandez, Zhu Wang, Fabiola Balarezo, Jeffrey S Hyams, Francisco A Sylvester, Anthony T Vella

**Affiliations:** 1 Department of Pediatrics, Division of Digestive Diseases, Hepatology & Nutrition, Connecticut Children's Medical Center, Hartford, CT, USA; 2 Department of Immunology, University of Connecticut Health Center, Farmington, CT, USA; 3 Department of Pediatrics, Division of Gastroenterology, University of North Carolina School of Medicine, Chapel Hill, NC, USA; 4 Department of Research, Connecticut Children's Medical Center, Hartford, CT, USA; 5 Department of Pathology and Laboratory Medicine, Hartford Hospital, Hartford, CT, USA

## Abstract

Eosinophilic esophagitis (EoE) is an emerging allergic, IgE- and non-IgE (Th2 cell)-mediated disease. There are major gaps in the understanding of the basic mechanisms that drive the persistence of EoE. We investigated whether esophageal biopsies from children with EoE demonstrate an inflammatory response that is distinct from normal controls. We prospectively enrolled 84 patients, of whom 77 were included in our analysis, aged 4–17 years (12.8±3.8 years; 81% males). Five esophageal biopsies were collected from each patient at the time of endoscopy. Intramucosal lymphocytes were isolated, phenotyped and stimulated with phorbol 12-myristate 13-acetate/ionomycin to measure their potential to produce cytokines via flow cytometry. We also performed cytokine arrays on 72-h biopsy culture supernatants. CD8^+^ T cells, compared with CD4^+^ T cells, synthesized more TNF-α and interferon (IFN)-γ after mitogen stimulation in the EoE-New/Active vs EoE-Remission group (*P*=0.0098; *P*=0.02) and controls (*P*=0.0008; *P*=0.03). Culture supernatants taken from explant esophageal tissue contained 13 analytes that distinguished EoE-New/Active from EoE-Remission and Controls. Principal component analysis and cluster analysis based on these analytes distinctly separated EoE-New/Active from EoE-Remission and Controls. In summary, we have identified a previously unappreciated role for CD8^+^ T lymphocytes with potential to produce TNF-α and IFN-γ in EoE. Our results suggest that CD8^+^ T cells have a role in the persistence or progression of EoE. We have also identified a panel of analytes produced by intact esophageal biopsies that differentiates EoE-New/Active from EoE-Remission and controls. Our results suggest that esophageal epithelial cells may have specific immune effector functions in EoE that control the type and amplitude of inflammation.

Eosinophilic esophagitis (EoE) is an allergic, immune-mediated, clinicopathologic entity that is challenging to treat in pediatric and adult patients. To date, there are major gaps in the understanding of the basic mechanisms that drive the persistence of EoE. These gaps limit our ability to non-invasively diagnose and monitor the results of therapy in EoE patients. Although eotaxin-3 was identified as the most highly expressed chemokine in esophageal biopsies from patients with EoE,^1^ serum eotaxin-3 has low sensitivity and specificity to diagnose or monitor EoE.^1, 2, 3^ Dellon *et al.*^4^ investigated the utility of serum biomarkers in EoE including IL-4, IL-5, IL-6, IL-9, IL-13, TGF-β, TNF-α, eotaxin -1, -2, -3, and thymic stromal lymphopoietin and found no difference in serum levels in EoE vs controls nor were they affected by therapy. Despite significant advances in our understanding of the genetic susceptibility, cytokine responses and characterization of the cells that infiltrate the esophagus in EoE, expert consensus is that ‘no biomarker or pathognomonic element has been identified that would eliminate the need for both symptoms and abnormal histology to make the diagnosis'.^5^ Therefore, biomarkers that distinguish EoE and that aid in diagnosing and monitoring the disease with high specificity are needed.

It is believed that EoE is mainly driven by a Th2 inflammatory response leading to esophageal eosinophilia.^6, 7^ Current EoE data suggest that Th2 cells are activated by ingested or inhaled antigens and generate chemotactic signals for eosinophils, in particular, interleukin (IL)-5 and IL-13 based on microarray gene expression studies in whole esophageal biopsies from patients with active EoE.^1, 3, 8, 9, 10, 11, 12, 13, 14^ Nevertheless, IL-5 blockade in patients with EoE did not resolve the clinical symptoms compared with placebo but decreased the number of infiltrating eosinophils,^15, 16^ suggesting that in addition to eosinophils, other cells may be involved in the persistence of EoE. Therefore, we set out to characterize the inflammatory response focusing on esophageal mucosal CD8^+^ T cells. We also used a nonbiased approach to measure cytokines and other proteins released by the esophageal biopsy explant cultures from patients with EoE and normal controls.

Our overarching hypothesis is that a defined set of cytokines and chemokines drive EoE persistence or progression by facilitating hyper-responsiveness to food and/or environmental antigens through T-cell stimulation. This results in innate cell activation and ensuing damage to esophageal epithelial cells. We demonstrate specific differences in CD3^+^CD8^+^ T-cell cytokine potential in EoE, and importantly, also a distinctive cytokine/chemokine panel contained within patient biopsy explant cultures that reliably separate newly diagnosed EoE or active treated EoE from patients with inactive EoE or controls.

## RESULTS

### Patient demographics and clinical information

We prospectively enrolled 84 patients between 18 December 2011 and 28 August 2014; 77 were included in our analysis (mean age 12.8±3.8 years, 62 (81%) males): EoE-New/Active (*n*=28), EoE-Remission (*n*=24) and normal controls (*n*=25). We excluded four subjects from the analysis because of comorbidities (two with *Helicobacter pylori* gastritis, one with Barrett's esophagus and one with celiac disease), and in three subjects, only blood samples but no biopsies were available. The demographic, clinical, histological and endoscopic characteristics of all patients are listed in Table 1. Patients were enrolled either during their first endoscopy (8 EoE-New and 25 Controls) or repeat endoscopy (20 EoE-Active and 24 EoE-Remission).

There was no statistically significant difference in age, gender or body mass index between groups. Patients with EoE were more likely to have atopic disease (*P*<0.0001), elevated serum Immunoglobulin (Ig)E (*P*=0.0166) and higher peripheral eosinophil counts (*P*<0.0001) when compared with controls. Patients with EoE-New/Active were also more likely to have characteristic endoscopic findings (furrows, trachealization/rings, white specks, stricture) (*P*<0.0001) than patients with EoE-Remission and Controls. In addition, histologically, children with EoE-New/Active were more likely to have higher esophageal mucosal intraepithelial eosinophil counts (*P*<0.0001), severe basal layer hyperplasia (*P*<0.0001), papillary elongation (*P*<0.0001), superficial microabscesses (*P*<0.0001) and eosinophil degranulation (*P*<0.0001).

Most of the EoE-Active patients who failed to respond to treatment had been prescribed a proton pump inhibitor (PPI; 16 of 20) for a minimum of 4–12 weeks in addition to dairy elimination diet or six food elimination diet (14 of 20) and/or swallowed steroids (11 of 20) for a minimum of 8–12 weeks. Nine of 20 patients were on all three therapies (PPI, swallowed steroids and dietary elimination). The EoE-Remission patients were mainly on dietary elimination therapy (20 of 24 patients on six food elimination diet or dairy elimination) or swallowed steroids (4 of 24)±PPI therapy (18 of 24).

Patients who were randomly selected for the multiplex cytokine analysis were a fair representation of the overall cohort. Patient demographics and clinical information for this subset of patients are summarized in Table 2. Prior to the endoscopy, the patients were treated as follows: EoE-New patients (*n*=5) were not on any treatment prior to their endoscopy; EoE-Active patients (*n*=5) were on PPI therapy (*n*=4), dairy elimination (*n*=2), six food elimination diet (*n*=2) and/or swallowed steroids (*n*=2); EoE-Remission patients (*n*=5) were on on PPI therapy (*n*=4), dairy elimination (*n*=1), six food elimination diet (*n*=3), directed elimination (*n*=1) and/or swallowed steroids (*n*=1); and non-inflammatory controls (*n*=5) were not on any treatment except H2 blocker (*n*=1).

### Esophageal mucosal biopsies show no difference in the number of lymphocytes but possess differential cytokine potential among groups

**Figure 1:**
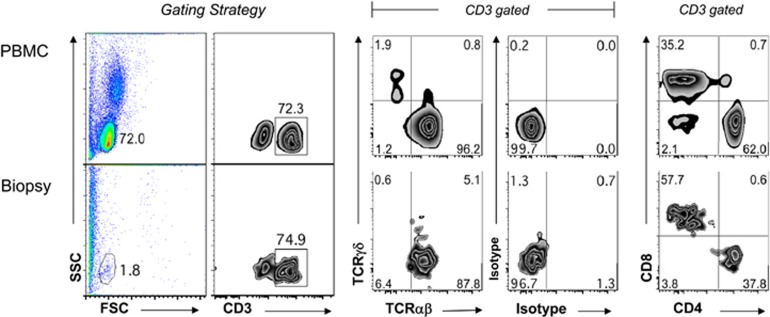
Flow cytometry gating strategy of cells isolated from esophageal biopsies: peripheral blood mononuclear cells were utilized to establish the lymphocyte gate (top panels). Flow cytometry plots representative of peripheral blood mononuclear cells and cells extracted from the biopsies are shown (bottom panels). Cells were identified as lymphocytes based on their forward (FSC) and size scatter (SSC) and analyzed for their expression of TCRαβ, TCRγδ, CD3, CD4 and CD8.

An average of 452 004±54 732 cells (range 140 000–1 264 006) were isolated from four enzymatically digested biopsies from each patient with no difference among the study groups. This is a confirmation that our biopsy collection and cell extraction methods were well controlled and consistent from patient to patient. Peripheral blood mononuclear cells from a normal control were utilized to establish the lymphocyte gate (Figure 1). The forward and size scatter allowed us to isolate the viable lymphocytes in the lymphocyte gate and exclude epithelial cells, dead cells and other cells present in the biopsies. Figure 1 shows the flow cytometry gating strategy and the major lymphocyte phenotypes present in the groups. Overall, across all groups, lymphocytes accounted for 4.4±3.4% (range 0.6–13.7%) of the total cell count. The average number of lymphocytes isolated was 14 984±1705, which was not significantly different among the groups. Flow cytometry data are summarized in Table 3. Although there was a significantly higher percentage of CD3^+^ lymphocytes in the EoE-New/Active group compared with the EoE-Remission (*P*=0.0047) and Control groups (*P*=0.0199), we did not detect significant differences in the total number of CD3^+^ T cells, percent or absolute number of CD3^+^CD4^+^ T cells, or percent or absolute number of CD3^+^CD8^+^ T cells.

**Figure 2:**
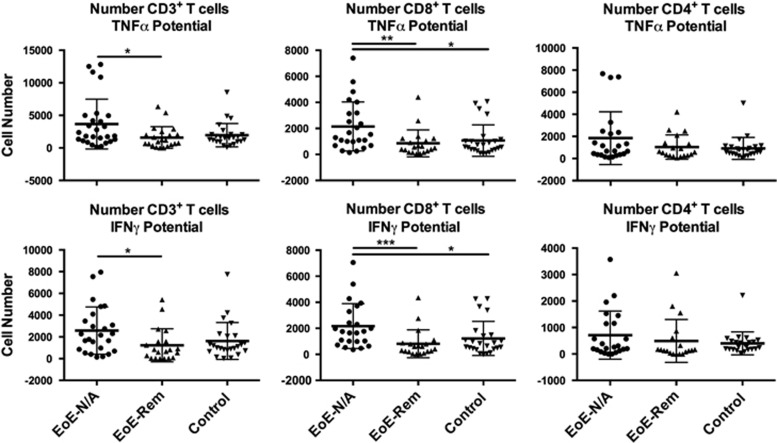
The potential of esophageal mucosal CD3^+^, CD3^+^CD8^+^ and CD3^+^CD4^+^ T lymphocytes to produce TNF-α or IFN-γ after phorbol 12-myristate 13-acetate/ionomycin stimulation was measured using flow cytometry. Graphic representation of the absolute number of CD3^+^, CD8^+^ and CD4^+^ T cells with potential to produce TNF-α and IFN-γ. There was no difference in TNF-α and IFN-γ potential in CD3^+^CD4^+^ cells (Table 3). *P<0.05, **P<0.01.

We measured the potential to secrete cytokines by lymphocytes isolated from esophageal mucosal biopsies by intracellular staining after phorbol 12-myristate 13-acetate/ionomycin stimulation or no stimulation for 4 h in the presence of BFA. We did not detect a difference in IL-5 or IL-13 production by CD3^+^, CD3^+^CD4^+^ or CD3^+^CD8^+^ T cells among the groups (data not shown). There was a significantly higher number of stimulated CD3^+^CD8^+^ T cells in the EoE-New/Active group that secreted TNF-α and interferon (IFN)-γ compared with the EoE-Remission and Control groups (Figure 2 and Table 3). There was no difference in secretion of TNF-α and IFN-γ by CD3^+^CD8^+^ T cells in the EoE-Remission and control groups. There was no difference in secretion of TNF-α or IFN-γ by the CD3^+^CD4^+^ T cells among all the groups (Figure 2 and Table 3).

### Delineating the inflammatory response in the esophagus in EoE patients

**Figure 3:**
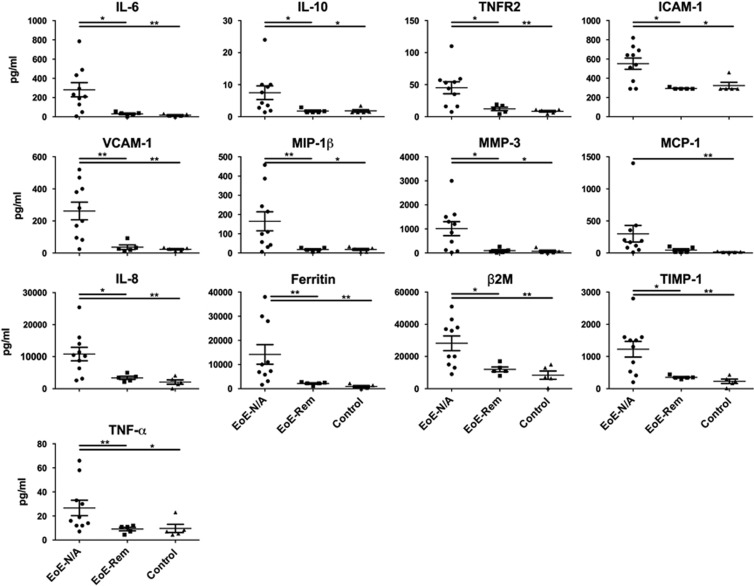
Esophageal explant culture supernatant analytes with differences among study groups. Supernatants were analyzed via multiplex cytokine analysis (Myriad RBM). Analytes with significant differences among the groups are shown. Kruskal–Wallis test followed by Dunn's multiple comparisons test was used to identify if there was a significant difference among the groups. *P<0.05, **P<0.01.

To study the continuation of the inflammatory response in the esophagus, we took esophageal biopsy samples and placed them in tissue culture without adding any known immunological stimuli. This approach allowed us to directly capture the continuation of the inflammatory response from the patients' esophagus *ex vivo*. Using biopsy culture supernatants, we determined the concentration of cytokines and chemokines in the study groups. The Myriad RBM Human Inflammatory Map 1.0 measures 45 analytes. The analytes were separated into three groups: undetectable (below the lowest detectable level), detectable but not significantly different among groups (has a measurable value above the lowest detectable level but with no significant difference among groups) and detectable with significant differences among groups (Table 4). Only 24 of 45 analytes had detectable levels, of which, 13 were found to have a statistically significant difference among the three groups (Figure 3 and Table 5). The significant analytes were β-2-microglobulin (B2M), ferritin, intracellular adhesion molecule-1 (ICAM-1), IL-6, IL-8, IL-10, monocyte inflammatory protein-1 beta (MIP-1β), matrix metalloproteinase-3 (MMP-3), monocyte chemotactic protein-1 (MCP-1), tissue inhibitor of metalloproteinase 1 (TIMP-1), tumor necrosis factor-alpha (TNF-α), TNF receptor-2 (TNFR2) and vascular adhesion molecule-1 (VCAM-1). Interestingly, 13 of 13 analytes were significantly higher in the EoE-New/Active group compared with the EoE-Remission and Control groups with no differences between the EoE-Remission and Control groups (Table 5).

### Principal component, cluster and pathway analysis

**Figure 4:**
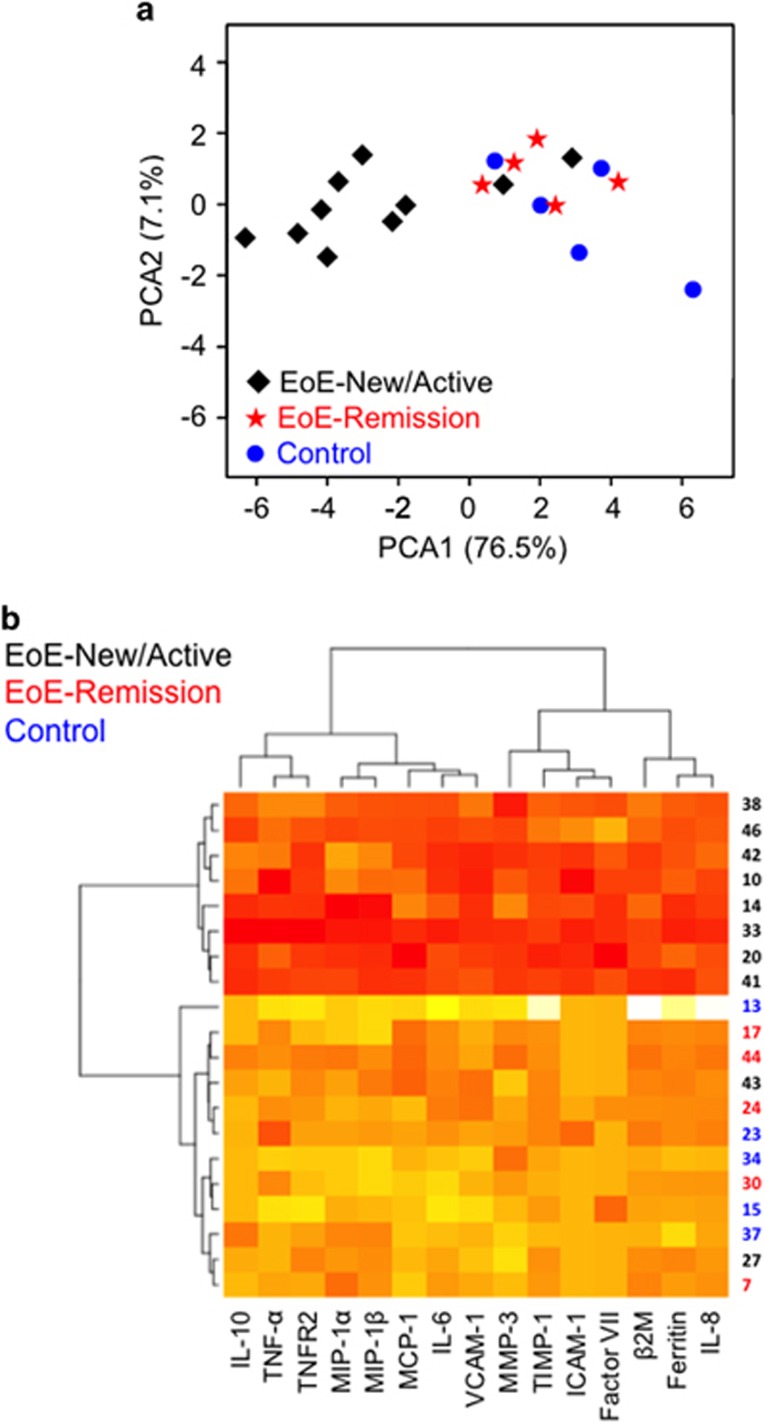
Patient group separation based on the cytokine network. (**a**) Unsupervised PCA of EoE-New/Active, EoE-Remission and Controls based on multiplex cytokine analysis from 72 h culture of esophageal biopsies. Two-dimensional PCA mapping represented 83% of variance (PC1=76% and PC2=7%). Each number represents a patient and patient groups are color-coded. (**b**) Unsupervised cluster analysis using cytokine levels between patient groups. Individual squares represent the cytokine concentration for the given cytokine (column) in a patient (row), with orange indicating higher cytokine levels and yellow indicating lower cytokine levels.

The two-dimensional principal component analysis (PCA) based on the analytes separated the EoE-New/Active patients from the EoE-Remission and Control groups (Figure 4a). The first and second principal components represented 83.6% of variance with the first principal component accounting for 76.5% and the second principal component accounting for 7.1% of the variance. Interestingly, 2 of 10 EoE-New/Active patients clustered with the other groups. These two patients were on montelukast for asthma. This raises the possibility that montelukast may have anti-inflammatory effects in EoE.

Unsupervised cluster analysis using analyte protein levels between patient groups showed clear grouping of EoE-New/Active while the other groups were scattered in the plot (Figure 4b).

According to Ingenuity Pathway Analysis (IPA), this panel of analytes found in EoE-New/Active patients centered around TNF-α and fit into known pathways that are involved in various functions including: hematologic system development and function (*P*=2.18 × 10^−16^), immune cell trafficking (*P*=1.77 × 10^−17^), inflammatory response (*P*=2.18 × 10^−16^), cellular movement (*P*=1.77 × 10^−17^), cell-to-cell signaling (*P*=2.18 × 10^−16^) and tissue development (*P*=4.57 × 10^−16^). The pathways predict involvement of various cell types including phagocytes, granulocytes and lymphocytes. Interestingly, this panel of analytes also resembled pathways involved in response to bacterial and viral infections (*P*=1.39 × 10^−13^).

## DISCUSSION

Prior to our study, cytokine/chemokine expression in EoE had been mainly based on mRNA abundance in whole biopsy extracts and immunohistochemical staining for pre-defined targets. Although measuring inflammatory biomarkers at the mRNA level is reliable in making the diagnoses and monitoring disease activity,^17^ knowing protein levels will add to the functional role immune cells have in EoE. We sought to approach biomarker mining in EoE from a different perspective by utilizing standard immunohistochemical and *ex vivo* techniques including flow cytometry and biopsy explant cultures.

First, utilizing flow cytometry, we identified a previously unappreciated role for CD3^+^CD8^+^ T cells in EoE patients. The CD3^+^CD8^+^ T cells had higher potential than CD3^+^CD4^+^ T cells to secrete TNF-α and IFN-γ in the EoE-New/Active vs EoE-Remission, suggesting that response to treatment leads to downregulation of TNF-α and IFN-γ production to levels as seen in controls. While we saw an increase in the number of CD3^+^CD8^+^ T lymphocytes and a higher CD8^+^/CD4^+^ ratio in the EoE-New/Active group, we did not appreciate a statistically significant increase in CD8^+^ or CD4^+^ populations suggesting a possible unexplored population of CD3^+^CD4^−^CD8^−^ T cells accumulating in the EoE-New/Active patients (compared with EoE-Remission patients and controls) that remains to be explored (Table 3).

While IL-5 and IL-13 production was inconsistent among our patients, we did not detect a statistically significant difference in IL-5 or IL-13 production by CD4^+^ T cells among the groups, suggesting an unappreciated role for TNF-α and IFN-γ producing CD8^+^ T cells in the progression or persistence of EoE inflammation. Lucendo *et al.*^18^ have previously demonstrated the predominance of CD8^+^ T cells in EoE via stereological microscopy but did not check for Tc1 cytokines. Krug *et al.*^19^ also demonstrated that while asthma is characterized by Th2 inflammatory response, IL-4 production was confined to a relatively small proportion of airway and blood T cells and there was selective enhancement of IFN-γ production by airway T cells. This might explain why we did not detect a difference in IL-5 or IL-13 production by stimulated T cells.

Our flow cytometry data demonstrate a role for CD8^+^ T cells with a Th1 response and a potential role for monocytes/dendritic cells in EoE. The pro-inflammatory cytokines TNF-α and IFN-γ have been shown to be involved in early inflammation as well as attenuation of inflammation in allergic and inflammatory disorders. IL-4 has been shown to be the principal stimulating factor for CCL26/eotaxin-3.^20^ TNF-α and IL-1β alone did not induce CCL26 expression, yet these pro-inflammatory cytokines synergized with IL-4 to increase CCL26 protein expression.^21^ Co-incubation of IFN-γ with IL-4 had no effect on CCL26 protein release. By contrast, pretreatment of human monocytes with IFN-γ decreased total STAT6 protein, blocked IL-4-mediated STAT6 phosphorylation and decreased IL-4-mediated CCL26 mRNA expression and protein release. These data show that IL-4 and pro-inflammatory cytokines such as TNF-α, IL-1β and IFN-γ regulate CCL26 synthesis in human monocytic cells, which may be important in regulating monocyte inflammatory responses.^21^ These data reaffirm our findings that TNF-α may have a role in the progression or persistence of EoE.

Second, the quantitative, multiplexed immunoassays of esophageal biopsy explant culture supernatants allowed us to identify a panel of analytes that may contribute to or explain EoE pathogenesis. Our PCA (Figure 4a) and hierarchical clustering (Figure 4b) analyses suggest that the panel of analytes can be the basis for new diagnostics that reliably distinguish EoE-New/Active from EoE-Remission and normal controls. Moreover, we believe that therapies can be designed to uncouple this panel of analytes leading to the successful treatment of EoE.

An intriguing question regarding the analytes is whether these factors promote or prevent inflammation and pathology. Clearly, the analytes track with disease activity as seen in the EoE-Remission group. We propose that these analytes may be primarily derived from the activated esophageal epithelium, owing to the overrepresentation of epithelial cells compared with lymphocytes in biopsies. Second, we found evidence for immune-regulatory factors such as TNFR2 and IL-10. It is striking that the analytes are mainly composed of innate immune-derived proteins, which contrasts from adaptive cytokines such as IFN-γ, IL-4, IL-5 and IL-13, which were not present or significantly different among the groups. Although we found that stimulated CD3^+^CD8^+^ T cells have increased IFN-γ in esophageal mucosal biopsies from EoE-New/Active (Figure 2b), IFN-γ was not detectable and not different among the study groups when we analyzed the supernatants (without stimulation) from explant tissue cultures (data not shown). Although eotaxin-1, IL-5 and IFN-γ were not detected or were not found to be statistically significant among the groups, it is possible that these analytes were produced but not detected by the technology. It is also possible that the immune cells require stimulation before these cytokines could be released. Therefore, it remains possible that these analytes are produced in EoE as seen in other studies.

**Figure 5:**
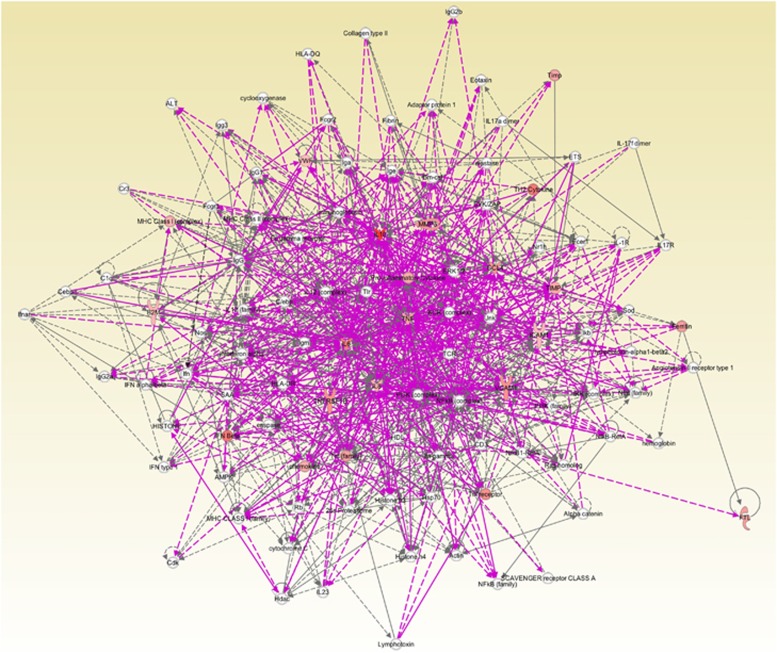
Pathway analysis using IPA based on the concentration of analytes in esophageal mucosal biopsy culture supernatants. We input the log2 ratio and *P* value of the difference between means of 13 factors that were different between EoE-New/Active and Normal controls into IPA software. The figure demonstrated the vast and complex interaction of the cytokines identified and how these cytokines revolve around TNF-α.

As seen by the IPA, this panel of analytes found in EoE-New/Active patients, revolves around TNF-α (Figure 5) and fits into known pathways that are involved in various functions including: hematologic system development and function, immune cell trafficking, inflammatory response, cellular movement, cell-to-cell signaling and tissue development. Interestingly, the IPA demonstrated that this panel of analytes also resembled pathways involved in response to bacterial and viral infections. This may be an indication that exposure to bacterial or viral infections may have a role in the initiation of EoE by disrupting the esophageal epithelial barrier, thus allowing food antigens to penetrate the epithelial layer leading to inflammation.

From a clinical perspective, the analytes may serve to diagnose and monitor disease activity in EoE patients and to develop new targeted diagnostics and therapies. This is important because there is no clear diagnostic test and there are few therapies available to treat EoE. Therapeutic options are limited to PPIs, swallowed fluticasone or budesonide, and severe dietary restriction or elemental formulas.

The pathogenesis of EoE has proven to be much more complex than just eosinophils and Th2 response. Once antigens are recognized by dendritic cells and Th2 lymphocytes, several cytokines/chemokines including eotaxin-3, IL-5 and IL-13 are released leading to cell recruitment and proliferation. Over the last decade, there have been many new discoveries in the field that have highlighted additional factors and cells involved in EoE. Cells including epithelial cells, eosinophils, mast cells, fibroblasts, basophils, lymphocytes and dendritic cells have been shown to have various roles in the pathogenesis of EoE. The thymic stromal lymphopoietin-Basophil response/pathway has been shown to contribute to the pathogenesis of EoE based on rodent and human studies.^22, 23^ Milk sphingolipids have shown to activate peripheral iNKT cells in EoE in children to produce Th2-type cytokine response.^24^ In addition, iNKT cell-associated markers were found to be upregulated in patients with EoE and correlated with the expression of inflammatory mediators associated with allergy. These findings were also more pronounced in patients <6 years of age.^25^ FOXP3^+^ regulatory T cells and CD8^+^ T cells have been shown to be increased in esophageal biopsies in EoE and GERD suggesting a possible negative mechanism that regulates the inflammatory response.^26, 27^ In addition, it has recently been shown that IL-18 and its receptor IL-18Rα are increased in the blood and esophagus, respectively, in patients with EoE. IL-18 stimulates iNKT cells and endothelial cells leading to induction of EoE cytokines IL-5 and IL-13.^28^ Finally, it has also been suggested that EoE in adults is likely an IgG4-associated disease and not an IgE-induced allergy based on failure of omalizumab (anti-IgE recombinant DNA-derived humanized IgG1k monoclonal antibody) to alter symptoms of EoE compared with placebo.^29^ Our study demonstrates that a single biomarker may not be sufficient to diagnose or monitor EoE and rather, a biomarker panel or network may be the way of the future.

**Figure 6:**
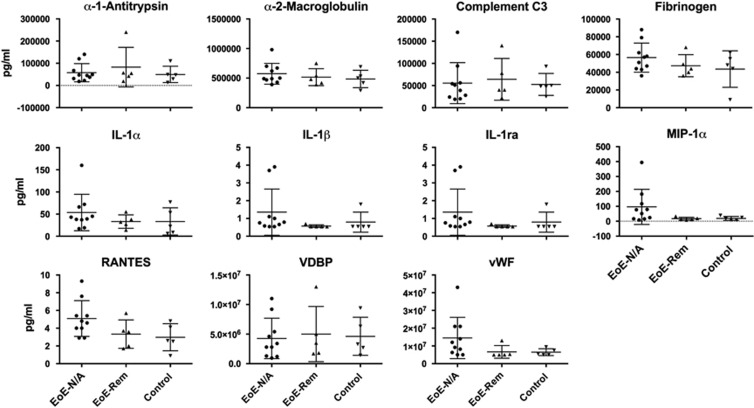
Esophageal explant culture supernatant analytes with no significant differences among study groups. Supernatants were analyzed via multiplex cytokine analysis (Myriad RBM). Analytes without significant differences among the groups are shown. Kruskal–Wallis test followed by Dunn's multiple comparisons test was used to identify if there was a significant difference among the groups. *P<0.05, **P<0.01.

There are many strengths to our study. This was a prospective study performed on human tissue obtained at the time of endoscopy and processed immediately. We have a large number of patients enrolled in the study with four different groups of patients (EoE-New, EoE-Active, EoE-Remission and Controls). We also utilized standard immunohistochemical techniques as well as *ex vivo* techniques to delineate the inflammatory response in EoE. A limitation of our study is perhaps the use of a single biopsy for our biopsy explant cultures. Some may argue that a single biopsy is too small and may not represent the inflammatory response in the entire esophagus. In addition, even in the presence of growth factors and serum, the cells likely have begun apoptosis, releasing cellular contents including cytokines and chemokines. We submit, however, under the same conditions, that our approach clearly distinguished patients with active inflammation from those in remission and normal controls (Figures 4a and 6b). In addition, there were several analytes that were either undetectable or had similar levels among the groups, acting as internal controls, which confirms that these analytes are strictly expressed in patients with active EoE. A longitudinal study and further validation of these analytes are warranted. The absence of secretion of IL-5 and IL-13 is possibly due to technical issues. However, it is also possible that these cytokines are produced elsewhere or confined to a small proportion of esophageal T cells.

In summary, we suggest that innate immune factors released by the esophageal epithelium upon activation by dietary antigens contribute to the pathogenesis of EoE. Our panel of analytes could potentially help in diagnosing and/or monitoring EoE but are not definite. In addition, CD3^+^CD8^+^ T cells may become activated by these factors and secrete TNF-α and IFN-γ to perpetuate inflammation. Our work expands the mechanistic spectrum of EoE.

## METHODS

### Patients

We prospectively enrolled children between the ages of 4 and 17 years at the time of a medically indicated esophagogastroduodenoscopy at Connecticut Children's Medical Center (CCMC), Hartford, CT, between 18 December 2011 and 28 August 2014. Informed consent to participate in the study was obtained from the patients' parent/s and assent was obtained from patients ⩾7 years of age. Inclusion criteria included: (i) children with known EoE who underwent endoscopy after specific food reintroduction or after starting medication (typically oral budesonide 0.5–1 mg BID in a Splenda^30^ (Heartland Consumer Products, LLC, Carmel, IN, USA) or Duocal slurry (Nutricia North America, Gaithersburgh, MD, USA) or swallowed fluticasone oral puffs^31, 32^ for 8–12 weeks) and (ii) children undergoing esophagogastroduodenoscopy for suspected EoE based on clinical presentation (difficulty swallowing, pain on swallowing, food impaction, persistent reflux symptoms despite PPI therapy and vomiting). We excluded from the analysis children with comorbidities such as celiac disease, inflammatory bowel disease, connective tissue disorders and *Helicobacter pylori* gastritis. We collected demographic information (age, gender, smoking exposure), clinical information (body mas index, smoking exposure and history of atopic disease), endoscopic findings (furrows, white specks, trachealization and strictures) and histologic data (peak eosinophil counts and basal layer hyperplasia).

### Biopsies

During the endoscopy procedure, a total of five biopsies were collected from each patient (three biopsies from the lower-mid esophagus and two biopsies from the upper esophagus). Four biopsies were used for lymphocyte isolation and flow cytometry and one biopsy from the lower esophagus was used for culture. Patients with active EoE were only included if they had inflammation in both the upper and lower esophagus. Patients in remission and the normal controls had no inflammation in both the upper and lower esophagus. The biopsies were placed in tubes containing RPMI 1640, placed on ice in a Styrofoam box and transported to our laboratory at the University of Connecticut Health Center. We obtained a complete blood count with differential, sedimentation rate (erythrocyte sedimentation rate) and a serum IgE level during intravenous line insertion at the time of endoscopy.

### Patient groups

A diagnosis of EoE was confirmed, according to the 2011 EoE consensus statement^33^ and was based on clinical history (typical symptoms and failure to respond to PPI therapy), endoscopic (abnormal endoscopic findings: furrows, white specks, stricture, trachealization) and histologic findings (⩾15 eosinophils per high-powered field (eos/hpf), basal layer hyperplasia and papillary elongation). We defined three study groups as follows: (i) EoE-New/Active: patients with histologically active disease (⩾15 eos/hpf in the lower and upper esophagus)—untreated/newly diagnosed or treated/known EoE who underwent endoscopy after treatment with either specific food reintroduction post elimination diet for 8–12 weeks or after receiving medication (budesonide or fluticasone propionate) for 8–12 weeks and were found to have persistence of esophageal eosinophilia (⩾15 eos/hpf); (ii) EoE-Remission: patients with successfully treated disease—known EoE, inflammation resolved after treatment with elimination diet or swallowed steroids (<5 eos/hpf in the lower and upper esophagus); and (iii) Controls: patients who underwent esophagogastroduodenoscopy for evaluation of dysphagia, odynophagia, suspected reflux or EoE and who were found to have no visual (endoscopic) or histologic evidence of esophageal inflammation (<1 eos/hpf). We decided to group together the EoE-New and EoE-Active because of their demographic, clinical, endoscopic, histologic and immunologic similarities.

### Esophageal mucosal cell isolation and preparation

Biopsies were obtained with the same model forceps in all patients. We followed previously published methods used in our laboratory for cell isolation with slight modifications for the esophageal epithelium.^34, 35^ Briefly, four biopsies from each patient (two from the lower and two from the upper esophagus) were enzymatically digested in pre-warmed HBSS with CaCl_2_ and MgCl_2_ solution (Life Technologies, Grand Island, NY, USA) containing 150 U ml^−1^ collagenase from *Clostridium histolyticum* (Sigma-Aldrich, St Louis, MO, USA), 100 μg ml^−1^ dispase II from *Bacillus polymyxa* (Roche, Indianapolis, IN, USA) and 0.1 mg ml^−1^ DNAse I (Sigma-Aldrich) for 30 min at 37 °C, spinning at 450 r.p.m. The digested tissue was filtered through a 70-μm nylon mesh cell strainer (BD Biosciences, San Diego, CA, USA). The remaining tissue in the strainer was mashed through the cell strainer and washed with culture media (RPMI 1640, Sigma-Aldrich) containing 1% L-glutamine, 10% fetal bovine serum, 1% non-essential amino acids, 1% sodium pyruvate, 1% antibiotics/antimycotic (Invitrogen, Carlsbad, CA, USA). Extracted cells were centrifuged at 1000 r.p.m., at 4 °C, for 5 min. The supernatant was discarded, and the pellet was resuspended in 1 ml of culture media. The isolated esophageal cells were counted using a Z1 Beckman Coulter Particle Counter (Beckman Coulter, Inc., Brea, CA, USA).

### Cell surface staining and flow cytometry

For each patient, we plated all cells extracted from esophageal biopsies in 96-well flat -bottomed plates (NEST Biotechnology, Shanghai, China). Peripheral blood mononuclear cells from normal volunteers were used as the control to generate gating strategy. The plates were centrifuged at 1000 r.p.m. for 3 min at 4 °C, and then the supernatants were discarded. After discarding the supernatant, cells were incubated for 30 min on ice in wash buffer with primary antibodies against anti-human anti-CD3-Vioblue (Mylteny Biotec, Inc., Auburn, CA, USA), anti-CD4-PerCP Cy5.5 (Biolegend, San Diego, CA, USA), anti-CD8-V500 (BD Biosciences), anti-TCRαβ-FITC (eBioscience, San Diego, CA, USA) or anti-CD11b-PE Cy7 (Biolegend) as described previously.^36^ Control cells were incubated with anti-mouse IgG1-allophycocyanin (eBioscience), anti-mouse IgG1-FITC (Biolegend), anti-rat IgG1-phycoerythrin (PE) (eBioscience), 1:5 dilution of anti-rat IgG2a-PE (eBioscience) or anti-mouse IgG1-PE Cy7 (Biolegend). The cells were then washed in wash buffer and resuspended in 200 μl of wash buffer and transferred into fluorescence-activated cell sorting tubes. Cells were analyzed with flow cytometry on FACS-LSRII (BD Biosciences), and data were analyzed using FlowJo software (Tree Star, Ashland, OR, USA).

### *In vitro* lymphocyte stimulation and intracellular staining

The cells were incubated with culture media containing 200 μg ml^−1^ brefeldin A (BD Biosciences) with or without stimulation with 1 × phorbol 12-myristate 13-acetate (Calbiochem, EMD Chemicals, Inc., Gibbstown, NJ, USA) and ionomycin (1 μg ml^−1^; Sigma, St Louis, MO, USA) for 4 h at 37 °C. The plate was then centrifuged at 1000 r.p.m. for 3 min at 4 °C. After surface staining with anti-human anti-CD3, CD4 and CD8, the cells were fixed and permeabilized for intracellular staining. Cells were incubated with anti-TNF-α-allophycocyanin (Biolegend), anti-IFN-γ-FITC (Biolegend) and IL-5-PE (eBioscience). Briefly, cells were washed in wash buffer and resuspended in 200 μl of wash buffer and transferred into fluorescence-activated cell sorting tubes to be analyzed with flow cytometry as described above.

### Biopsy explant culture

To study the inflammatory response present in the esophagus *ex vivo*, a single mucosal biopsy, from the lower esophagus, was obtained with standardized forceps. The biopsy was placed in 1 ml culture medium (RPMI 1640 with supplements as mentioned above including 10% fetal bovine serum) in 5% CO_2_ at 37 °C for 72 h. The culture was then centrifuged at 14 000 r.p.m., 4 °C for 3 min. The supernatants were removed, dispensed in aliquots and frozen at −80 °C until analyzed.

### Multiplex cytokine assay

The biopsy culture supernatants from 20 patient samples (5 EoE-New, 5 EoE-Active, 5 EoE-Remission and 5 controls) were sent to Myriad RBM (Austin, TX, USA) for quantitative measurement of 45 analytes (Human Inflammation Map1.0) utilizing microsphere-based immune-multiplexing assay on the Luminex platform.

### Pathway analysis

The cytokine levels were all converted to pg ml^−1^ and *log 10*-transformed to correct for skewed data (asymmetry of data in relation to the mean). We input the *log 10* ratios and *P* values of EoE-New/Active vs Controls into the online Ingenuity Pathway Analysis program (IPA, Ingenuity Systems, www.ingenuity.com) to find fits for our cytokine network in known pathways. The software performs global functional analysis in global canonical pathways to determine the *P* value for a function or pathway. The software utilizes right-tailed Fisher Exact Test to measure the likelihood that the association is due to random chance. The smaller the *P* value (*P*<0.05), the less likely that the statistical association is random and the more significant the statistical association is.

### Statistical analysis

Statistical analysis was performed using Prism Software version 6.0 (GraphPad Software Inc. La Jolla, CA, USA). Data were described using mean±s.d. or s.e.m. For the flow cytometry and multiplex cytokine analyses, we used nonparametric, Kruskal–Wallis tests followed by Dunn's multiple comparisons test. Results were considered statistically significant at *P*<0.05. We utilized the Mann–Whitney test (nonparametric) to determine statistical significance when comparing two groups. PCA using only those analytes selected from the *P*<0.05 level of significance was applied, and the resulting top two components were plotted in a two-dimentional plot. PCA reveals the internal structure of data in a way that best explains the variance. The hierarchical clustering method was used to group a set of study subjects in such a way that subjects in the same group are more similar in terms of analytes to each other than to those in other groups. The hierarchal, unsupervised cluster analysis and PCA were performed using R program (version 3.0.1) from R foundation for statistical computing (http://www.R-project.org).

## Figures and Tables

**Table 1:** Patient demographics and clinical information

	*EoE-N/A*	*EoE-Rem*	*Control*	P-*value*
Subjects (*n*=77)	28	24	25	
				
*Demographics*				
Age at time of endoscopy (years)	12.2±4	12.86±3.3	11.85±4.4	0.6451^a^
Gender, *n* (%)				
Female	4 (14)	2 (8)	9 (36)	0.0597^b^
Male	24 (86)	22 (92)	16 (64)	
Gender (all EoE vs Control)—*n* (%) Male	46 (88)		16 (42)	0.0177^b^
Ethnicity/Race				
White/Caucasian	20 (71)	21 (88)	20 (80)	0.5236^b^
Other	8 (29)	4 (12)	5 (20)	
BMI (kg m^−2^)	20.63±6.4	21.13±6.8	19.34±4.5	0.5279^a^
Atopic disease, *n* (%)	16 (57)	14 (58)	5 (20)	0.0088^b^
Smoking exposure, *n* (%)	4 (14)	5 (21)	1 (4)	0.2301^b^
				
*Laboratory tests*^c^				
Patients with elevated serum IgE, *n* (%)	13/19 (68)	9/14 (64)	5/18 (28)	0.0284^b^
Serum IgE (kU l^−1^)	407±395	277±309	139±236	0.0566^a^
Patients with elevated peripheral eosinophils, *n* (%)	7/21 (33)	2/16 (13)	0/18 (0)	0.0173^b^
Peripheral eosinophils (%)	8.9±4.7	4.9±4.2	3.1±1.9	<0.0001^a^
Absolute peripheral eosinophil count (eos ul^−1^)	536±367	201±229	161±88	<0.0001^a^
Patients with elevated ESR, *n* (%)	3/19 (16)	0/15 (0)	1/20 (5)	0.1906^b^
ESR (mm h^−1^)	9.84±9.86	8.13±4.57	10.11±4.8	0.6861^a^
				
*Endoscopic findings*				
Endoscopic findings (furrows, white specks, rings, strictures), *n* (%)	25 (89)	9 (38)	3 (12)	<0.0001^b^
				
*Histology*				
Eosinophils/hpf	44±23	3.6±4	0.1±0.3	<0.0001^a^
Eosinophil degranulation, *n* (%)	20 (71)	4 (17)	0	<0.0001^b^
Basal layer hyperplasia, *n* (%)				
None-mild	1 (4)	22 (92)	20 (80)	<0.0001^b^
Medium	7 (25)	2 (8)	5 (20)	
Severe	20 (71)	0	0	
Microabscesses, *n* (%)	12 (43)	0	0	<0.0001^b^
Papillary elongation, *n* (%)	28 (100)	10 (42)	3 (12)	<0.0001^b^

Abbreviations: BMI, body mas index; EoE, eosinophilic esophagitis; EoE-N/A, EoE-New/Active; EoE-Rem, EoE-Remission; ESR, erythrocyte sedimentation rate; hpf, high power field; IgE, immunoglobulin E.

Absolute values (age; serum IgE levels, absolute and percent peripheral eosinophil counts; absolute esophageal mucosal eosinophil counts per hpf and BMI) are expressed in mean±s.d.

a Kruskal-Wallis and *t*-tests for continuous data to compare groups.

b Contingency table and Chi square for comparison of categorical values.

c Laboratory tests were not performed on all patients.

**Table 2:** Demographics of patients selected for the multiplex cytokine analysis

	*EoE-N/A*	*EoE-New*	*EoE-Active*	*EoE-Rem*	*Controls*	P-*value*
Number (63)	10	5	5	5	5	
						
*Demographics*						
Age at time of endoscopy, mean±s.d.	12.4±3.9	11.97±4.7	12.8±3.4	14.97±1.8	13.5±1.99	0.7834^a^
Gender, *n* (%M)	8 (80)	5 (100)	3 (60)	4 (80)	2 (40)	0.1901^b^
BMI, mean±s.d.	23±9	20.8±6.3	25.1±11.52	24.3±8.6	23.9±6.7	0.8687^a^
						
*Clinical history*						
Atopic disease, *n* (%)	5 (50)	2 (40)	3 (60)	4 (80)	1 (20)	0.2615^b^
						
Laboratory tests*						
Patients with elevated serum IgE, *n* (%)	8 (80)	4 (80)	4 (80)	4 (80)	2 (40)	0.4142^b^
Serum IgE (kU l^−1^)	551±463	694±514	408±426	503±452	21±22	0.4071^a^
Absolute eosinophil count ul^−1^	394±217	402±146	385±311	288±236	58±66	0.0645^a^
ESR (mm h^−1^), mean±s.d.	11±8.7	10.6±8.7	11.5±10	7±3.4	8.7±2	0.8217^a^
						
*Endoscopic findings*						
Endoscopic findings (furrows, white specks, rings, strictures), *n* (%)		5 (100)	5 (100)	2 (40)	0	0.0018^b^

*Histology*						
Average Eos/hpf, mean±s.d.	54±28	50±25	57±33	2±2.7	0	0.0004^a^
						
*Treatment at time of endoscopy*						
PPI therapy	4 (40)	0	4 (80)	4 (80)	0	
H2 blocker	0	0	0	0	1 (20)	
Oral corticosteroids	2 (20)	0	2 (40)	1 (20)	0	
Dairy free diet	2 (20)	0	2 (40)	1 (20)	0	
SFED	2 (20)	0	2 (40)	3 (60)	0	
Directed elimination	0	0	0	1 (20)	0	
None	5 (50)	5 (100)			5 (100)	

Abbreviations: BMI, body mas index; EoE, eosinophilic esophagitis; EoE-N/A, EoE-New/Active; EoE-Rem, EoE-Remission; ESR, erythrocyte sedimentation rate; hpf, high power field; IgE, immunoglobulin E; PPI, proton pump inhibitor; SFED, six food elimination diet.

Absolute values (age; serum IgE levels, absolute peripheral eosinophil counts; absolute esophageal mucosal eosinophil counts per hpf; and BMI) are expressed in mean±s.d.

a Kruskal–Wallis and *t*-tests for continuous data to compare groups.

b Contingency table and Chi square for comparison of categorical values.

*Laboratory tests were not performed on all patients.

**Table 3:** Results of immunological analysis via flow cytometry

	*Subject groups*			*Statistics*			
	*EoE N/A (*n=*28)*	*EoE-Rem (*n=*24)*	*Control (*n=*25)*	P-*value*^a^	*EoE-N/A vs EoE-Rem* P-*value*^b^	*EoE-N/A vs Control* P^b^	*EoE-Rem vs Control* P-*value*^b^
Total cell number	452 004±54 732	463 159±47 251	307 620±38 074	0.022	>0.9999	0.0752	0.0341
% Lymphocytes	4.7±0.7	3.8±0.7	4.6±0.6	0.2438	0.6986	>0.9999	0.3062
Number of lymphocytes	16 948±3002	15 556±3881	12 235±1627	0.6449	>0.9999	>0.9999	>0.9999
% CD3^+^ lymphocytes	64.5±3.6	48.5±4.0	53.1±3.4	0.0024	0.0047	0.0199	>0.9999
Number CD3^+^ lymphocytes	11 429±2308	7400±1730	5989±841	0.213	0.4262	0.3697	>0.9999
% CD3+CD4^+^ lymphocytes	39.7±2.4	44.9±3.2	43.2±2.5	0.3418	0.445	>0.9999	>0.9999
Number CD3^+^CD4^+^ lymphocytes	4991±1069	3851±1163	2556±398	0.4477	0.9052	0.7654	>0.9999
% CD3^+^CD8^+^ lymphocytes	49.2±2.6	43.6±3.1	42.3±2.4	0.1962	0.3596	0.381	>0.9999
Number CD3^+^CD8^+^ lymphocytes	5307±1269	2725±433	2475±370	0.0478	0.1971	0.0611	>0.9999
CD8/CD4 ratio	1.6±0.2	1.3±0.3	1.1±0.1	0.2918	0.4465	0.6807	>0.9999
% CD3^+^ T cells with TNF-α potential	33.9±2.9	29.3±3.8	33.3±2.9	0.6408	>0.9999	>0.9999	>0.9999
Number CD3^+^ T cells with TNF-α potential	3667±738	1592±355	1957±361	0.0433	0.0401	0.3982	0.9478
% CD3^+^CD4^+^ T cells with TNF-α potential	28.9±3.2	38.5±3.1	36.7±2.9	0.0729	0.1035	0.2316	>0.9999
Number CD3^+^CD4^+^ T cells with TNF-α potential	1841±498	1034±252	914±201	0.5853	>0.9999	>0.9999	>0.9999
% CD3^+^CD8^+^ T cells with TNF-α potential	43.2±3.9	35.9±4.9	40.6±4.2	0.4544	0.6678	>0.9999	>0.9999
Number CD3^+^CD8^+^ T cells with TNF-α potential	2150±385	858±231	1068±242	0.005	0.0098	0.0247	>0.9999
% CD3^+^ T cells with IFN-γ potential	27.9±2.9	21.4±3.8	26.2±3.1	0.3925	0.5484	>0.9999	0.9844
Number CD3^+^ T cells with IFN-γ potential	2579±435	1224±340	1627±347	0.0167	0.0135	0.3115	0.3016
% CD3^+^CD4^+^ T cells with IFN-γ potential	15.0±2.6	17.2±3.3	17.3±2.1	0.5505	>0.9999	0.8244	>0.9999
Number CD3^+^CD4^+^ T cells with IFN-γ potential	711±189	492±186	401±90	0.179	0.3267	>0.9999	0.2914
% CD3^+^CD8^+^ T cells with IFN-γ potential	51.5±4.7	33.7±5.5	44.7±4.8	0.0739	0.0681	0.9491	0.5577
Number CD3^+^CD8^+^ T cells with IFN-γ potential	2175±358	811±239	1222±268	0.0009	0.0008	0.0332	0.6557

Abbreviations: EoE, eosinophilic esophagitis; EoE-N/A, EoE-New/Active; EoE-Rem, EoE-Remission; IFN, interferon; TNF-α, tumor necrosis factor-alpha.

Lymphocytes extracted from esophageal mucosal biopsies were analyzed as described in the legend of Figure 1. Values are in absolute cell numbers and presented with mean±s.e.m. We used nonparametric.

a Kruskal–Wallis tests followed by Dunn's multiple comparisons test.

b Mann–Whitney test (nonparametric) to determine statistical significance when comparing two groups. Results were considered statistically significant at *P<*0.05*.*

**Table 4:** Summary of undetectable, detectable and significant analytes

*Undetectable*	*Detectable but not significant*	*Detectable and significant*
Brain-derived neurotrophic factor (BDNF) C-reactive protein (CRP) Eotaxin-1 Factor VII Granulocyte-macrophage colony-stimulating factor (GM-CSF) Haptoglobin Interferon gamma (IFN-γ) Interleukin-2 (IL-2) Interleukin-3 (IL-3) Interleukin-4 (IL-4) Interleukin-5 (IL-5) Interleukin-7 (IL-7) Interleukin-12 subunit p40 (IL-12p40) Interleukin-12 subunit p70 (IL-12p70) Interleukin-15 (IL-15) Interleukin-17 (IL-17) Interleukin-18 (Il-18) Interleukin-23 (IL-23) Matrix metaloproteinase-9 (MMP-9) Stem cell factor (SCF) Tumor necrosis factor-beta (TNF-β) Vascular endothelial growth factor (VEGF)	Alpha-1-antitrypsin (AAT) Alpha-2-macrogloulin (A2Macro) Complement C3 (C3) Fibrinogen Interleukin-1 alpha (IL-1α) Interleukin-1 beta (IL-1β) Interleukin-1 receptor antagonist (IL-1ra) Macrophage inflammatory protein 1-alpha (MIP-1α) T-cell-specific protein RANTES (RANTES) Vitamin D-binding protein (VDBP) von Willebrand factor (vWF)	Beta-2-microglobulin (B2M) Ferritin Intracellular adhesion Molecule-1 (ICAM-1) Interleukin-6 (IL-6) Interleukin-8 (IL-8) Interleukin-10 (IL-10) Macrophage Inflammatory Protein 1 beta (MIP-1β) Matrix metalloproteinase-3 (MMP-3) Monocyte chemotactic protein 1 (MCP-1) Tissue inhibitor of metalloproteinase 1 (TIMP-1) Tumor necrosis factor-alpha (TNF-α) Tumor necrosis factor receptor-2 (TNFR2) Vascular cell adhesion molecule-1 (VCAM-1)

The Myriad RBM Human Inflammatory cytokine panel consisted of 45 analytes. Only 24 analytes were detectable, of which, 13 analytes were found to have a significant difference among the three groups. Definitions: *undetectable*—values are below the lowest detectable level; *detectable but not significant*—levels are above the lowest detectable level but show no difference among the groups; *detectable and significant*—levels are above the lowest detectable levels and show a difference among the groups.

**Table 5:** Cytokine concentrations in biopsy culture supernatants

*Analytes*	*Subject groups*			*Statistics*			
	*EoE N/A (*n=*10)*	*EoE-Rem (*n=*5)*	*Control (*n=*5)*	P-*value*^a^	*EoE-N/A vs EoE-Rem* P-*value*^b^	*EoE-N/A vs Control* P-*value*^b^	*EoE-Rem vs Control* P-*value*^b^
IL-6	281.5±73.7	30.6±8.8	16.2±5.5	0.0019	0.0127	0.008	0.2222
IL-10	7.5±2.1	1.76±0.3	1.8±0.4	0.0052	0.0143	0.0143	>0.9999
TNFR2	45.1±9.4	12.2±2.8	8.5±1.4	0.004	0.0373	0.004	0.3413
ICAM-1	552±59	294±4	324±34	0.0062	0.015	0.0223	>0.9999
VCAM-1	262±55	36.6±14	23.4±4.1	0.0013	0.008	0.0047	0.746
MIP-1β	165±50	17.6±4.1	18.3±5.3	0.0037	0.009	0.0123	0.9524
MMP-3	1014±291	94±45	74±43	0.008	0.028	0.0127	0.5317
MCP-1	300±129	46.2±20	15±3	0.0052	0.0539	0.005	0.2222
IL-8	10 833±2112	3386±501	2089±684	0.0016	0.0193	0.0047	0.2222
β2M	28 200±4572	12 000±3317	8390±2585	0.0038	0.0226	0.007	0.3492
Ferritin	14 170±4066	2140±277	924±393	<0.0001	0.0073	0.0013	0.0556
TIMP-1	1226±242	352±24	231±69	0.0023	0.0173	0.0073	0.1349
TNF-α	26.7±6.4	9.1±1.4	9.7±3.4	0.009	0.009	0.0306	0.627

Abbreviations: EoE, eosinophilic esophagitis; EoE-N/A, EoE-New/Active; EoE-Rem, EoE-Remission.

We analyzed 72-h biopsy culture supernatants by Myriad RBM. We present only analytes with significantly different concentrations among the groups and between subgroups (EoE-New/Active (N/A) vs EoE-Remission (Rem), EoE-N/A vs Control, and EoE-N/A vs GERD). Values are in pg ml^−1^ and presented with mean±s.e.m. We used nonparametric.

a Kruskal–Wallis tests (multiple or >2 groups) followed by Dunn's multiple comparisons test.

b The Mann–Whitney test (two groups) was used to compare EoE-New/Active with each of the other groups. Results were considered significant at *P*<0.05.
